# De novo protein design: a transformative frontier in clinical protein applications

**DOI:** 10.1186/s12967-026-07784-0

**Published:** 2026-02-04

**Authors:** Jie Gao, Zaiyong Zheng, Xueting Yu, Yamei Luo, Yang Yu, Chunxiang Zhang

**Affiliations:** 1 Department of Cardiology, The Affiliated Hospital, Southwest Medical University, No. 1, Section 1, Xianglin Road, Luzhou, Sichuan 646000 China; 2 Department of Cardiology, Panzhihua Central Hospital, Panzhihua, 617000 China; 3 Key Laboratory of Medical Electrophysiology, Ministry of Education & Medical Electrophysiological Key Laboratory of Sichuan Province, Institute of Cardiovascular Research, Southwest Medical University, Luzhou, Sichuan 646000 China; 4 Basic Medicine Research Innovation Center for Cardiometabolic Diseases, Ministry of Education, Southwest Medical University, Luzhou, Sichuan 646000 China; 5 Nucleic Acid Medicine of Luzhou Key Laboratory, Southwest Medical University, Luzhou, Sichuan 646000 China; 6 Chongqing University Jiangjin Hospital, Chongqing, 402260 China; 7 School of Medical Information and Engineering, Southwest Medical University, Luzhou, Sichuan 646000 China

**Keywords:** De novo protein design, Protein biologics, Translational medicine, Deep learning

## Abstract

**Background:**

Protein biologics are indispensable in disease prevention, diagnosis, and therapy, yet their development remains largely constrained by reliance on native protein scaffolds, resulting in long development timelines, limited structural and functional tunability, challenges in manufacturing consistency, and high production costs.

**Main body:**

De novo protein design moves beyond the structural and functional constraints inherent to traditional approaches, enabling the direct creation of proteins with tailored structures and functions and offering a new avenue to address these challenges. In this review, we summarize the principal computational strategies underlying de novo protein design and the contribution of deep learning to its recent progress, and highlight prospective applications, major translational barriers, and the current limitations and future challenges of the field.

**Conclusions:**

Despite notable methodological progress in de novo protein design, its path toward clinical application continues to be limited by a range of biological, technical, and translational considerations. Future work will need closer coordination between computational design, experimental validation, engineering optimization, and clinical needs, with clinical feasibility considered early and refined throughout development.

**Graphical Abstract:**

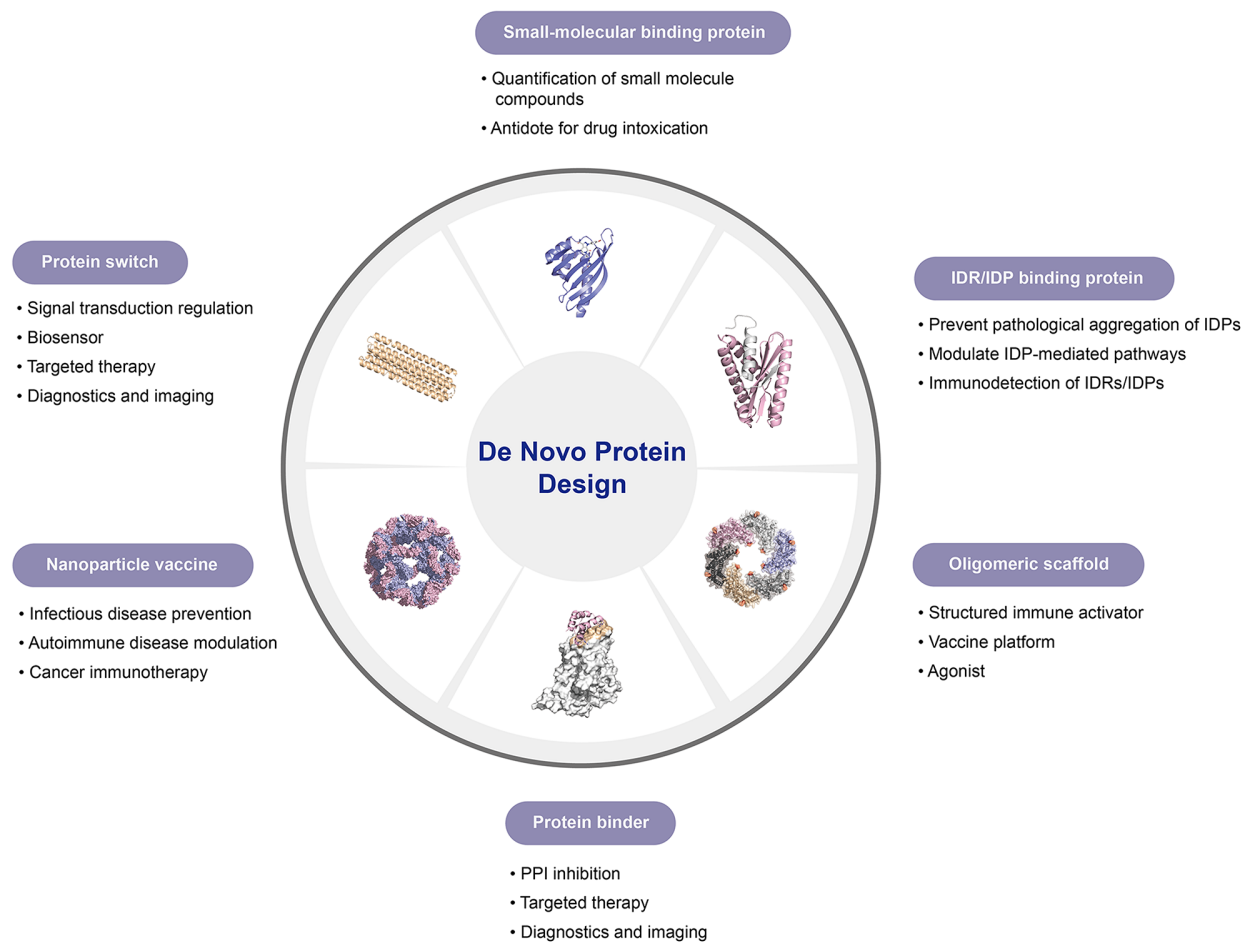

## Introduction

As essential components of life, proteins play a central role in nearly all biological processes. Protein-based diagnostics and therapeutics, particularly biologics, have become key tools in modern medicine. Since the introduction of recombinant human insulin in 1982 [1], protein biologics, especially monoclonal antibodies, have rapidly expanded to treat various diseases including cancer, inflammation, and infections [2–5]. In 2024 alone, over one-third of FDA-approved drugs were protein biologics [6]. However, their development continues to face considerable limitations and challenges.

In development, most protein biologics rely on laborious and time-intensive screening and optimization workflows [7]. For monoclonal antibodies, development begins with antigen production and animal immunization, followed by extensive screening through techniques such as hybridoma generation or phage display [8]. These processes rely heavily on the host immune system and offer limited human control. Similarly, synthetic biology-based strategies to modify natural proteins depend on iterative mutagenesis and high-throughput selection [9, 10]. These complex processes lead to high costs, long development cycles, significant batch-to-batch variability, and limited customizability [11].

Moreover, approximately 85% of protein targets associated with human diseases are commonly regarded as undruggable targets [12]. Many such undruggable targets contain intrinsically disordered regions or present functional interfaces that are excessively flat and expansive [13].

To overcome these challenges, de novo protein design has emerged as a promising alternative. De novo protein design refers to the design of entirely new protein structures and functions based on physical principles and computational methods, rather than direct modification of naturally evolved proteins [14]. This approach allows for rapid, cost-effective, and customizable protein biologics. In recognition of its groundbreaking impact, David Baker received the 2024 Nobel Prize in Chemistry for his work in de novo protein design, highlighting its promise for transforming biologic development [15].

While many excellent reviews on de novo protein design have emerged in recent years, their emphasis has largely been on the underlying algorithms and functionalities, with limited discussion of clinical translational potential [16–19]. Accordingly, this work integrates computational and clinical perspectives to bridge recent advances in de novo protein design with unmet needs in medical practice, and provides a structured discussion of potential applications, key barriers to clinical translation, and the current limitations and future challenges in this rapidly evolving field. To facilitate reader comprehension, relevant design methods and specialized terms are summarized in Table 1.

**Table 1:** Glossary of computational protein design methods and related terms

Term	Description
Rosetta	Rosetta is a physics-based protein design framework that predicts and optimizes protein structures by modeling atomic interactions. It emphasizes structural stability and energy minimization, but typically requires substantial computational resources and manual parameter tuning.
Hallucination	Hallucination refers to deep learning–based approaches that generate entirely new protein backbone structures without relying on natural protein templates, enabling exploration of novel protein folds not found in nature.
RFdiffusion	RFdiffusion is a diffusion-based generative model that generates new protein backbones under explicit structural or functional constraints, such as binding sites or functional motifs.
RFdiffusion All-atom	RFdiffusion All-atom extends RFdiffusion by incorporating atomic-level modeling, enabling protein design under constraints imposed by non-protein molecules, such as small-molecule ligands.
ProteinMPNN	ProteinMPNN is a deep learning–based sequence design tool that rapidly predicts amino acid sequences capable of stably folding onto a given protein backbone, significantly improving design efficiency and reducing experimental screening.
LigandMPNN	LigandMPNN extends ProteinMPNN by explicitly considering small-molecule ligands during sequence design, enabling the creation of proteins optimized for small-molecule recognition and binding.
Undruggable targets	targets that cannot be effectively modulated by conventional small-molecule or biologic therapeutics due to the absence of well-defined binding pockets or unfavorable physicochemical properties.
Chemistry, manufacturing, and controls (CMC)	Regulatory requirements describing the production process, quality control, and consistency of a therapeutic product to ensure safety and reproducibility in clinical use.
Directed Evolution	Random mutagenesis is used to artificially generate a diverse library of variants, which are subsequently screened against defined criteria to evolve proteins toward desired functions.
Non-antibody scaffolds	engineered protein frameworks distinct from immunoglobulin-based antibodies that can be designed or evolved to bind specific molecular targets with high affinity and specificity.
FcRn-mediated recycling	a cellular process in which the FcRn specifically binds and rescues IgG antibodies and albumin from lysosomal degradation, thereby markedly extending their circulating half-lives in vivo.
Non-residualizing / Residualizing labels	residualizing labels retain radioactivity within cells following internalization and degradation, whereas non-residualizing labels allow rapid efflux of radioactive catabolites, thereby reducing off-target tissue retention.
Immunotoxicity	adverse effects of a therapeutic agent on the immune system.
Exaggerated pharmacological effects	adverse effects arising from excessive or prolonged modulation of the intended target or pathway.
Binding-site barrier	a phenomenon in which high-affinity ligands are sequestered at accessible target sites near blood vessels, limiting further diffusion and penetration into deeper tissue regions.
Pharmacokinetics (PK)	the study of how a drug is absorbed, distributed, metabolized, and eliminated in vivo over time.
Pharmacodynamics (PD)	the study of the biological effects of a drug and the relationship between drug concentration and target engagement or functional response.
Minimum Anticipated Biological Effect Level (MABEL)	a dose-selection principle for first-in-human studies, defined as the lowest dose expected to elicit a measurable biological effect based on preclinical data.
Global backbone strain	the accumulation of unfavorable backbone geometry and torsional stress across the entire protein fold.
β-rich proteins	Proteins whose structures are dominated by β-sheet secondary elements.
β-strand swapping	β-strands from different monomers (or symmetric parts of the same monomer) interweave to form a shared, mixed β-sheet.

## Approaches of de novo protein design

**Fig. 1:**
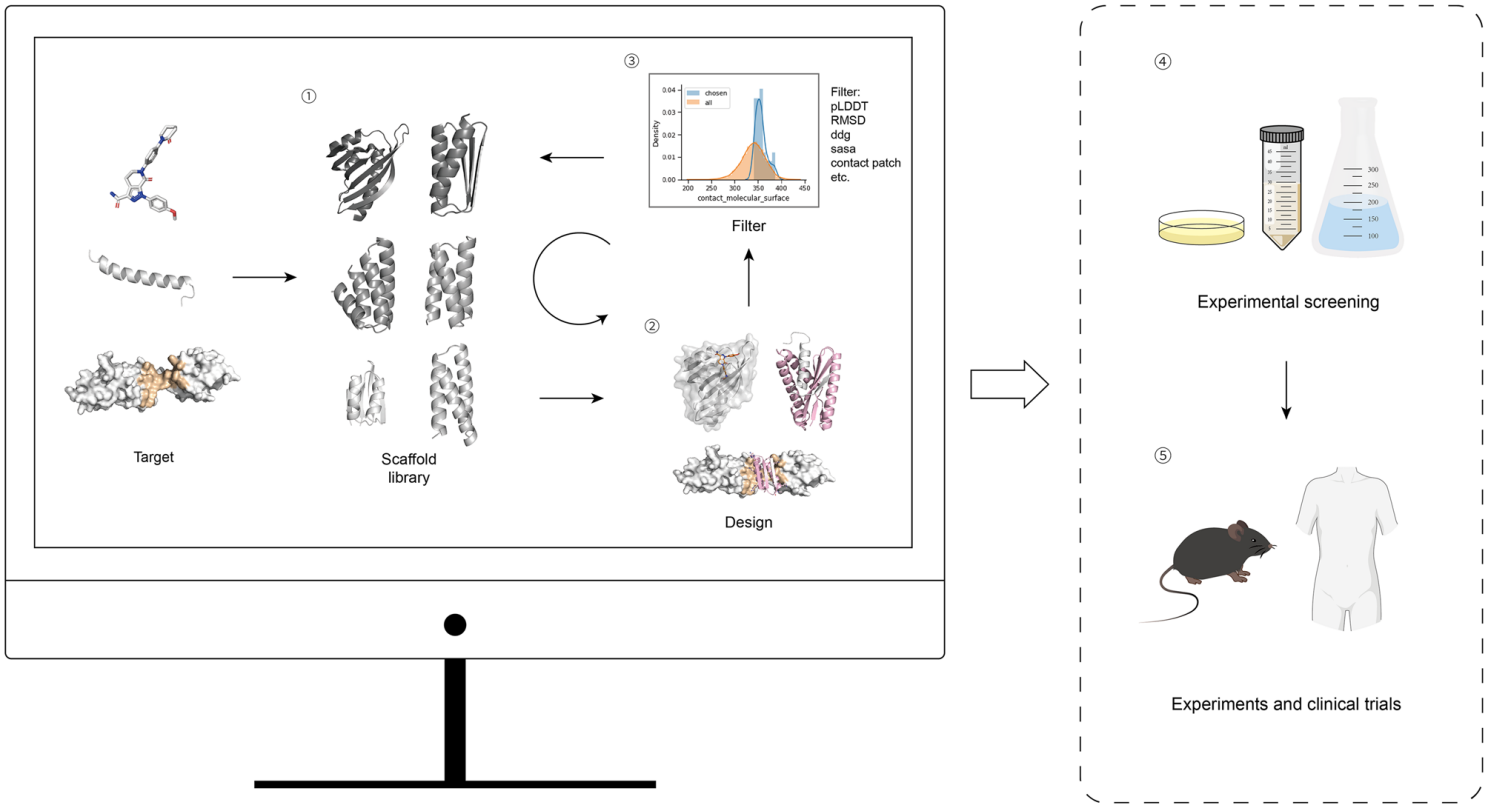
Overview of the de novo protein design workflow. The structures shown in steps ① and ② are sourced from [31, 40, 129] and the PDB database. Structural illustrations are produced with PyMOL [206]

Structure-based de novo protein design involves three main steps: backbone construction, sequence optimization, and candidate evaluation [20, 21]. This workflow remains widely used in binder design for protein interfaces, small molecules and peptides (Fig. 1).

The initial step involves constructing the tertiary structure of the protein backbone, which serves as a scaffold for design, commonly using algorithms like Hallucination [22], RFdiffusion [23], and RFdiffusion All-atom [24]. Certain open-source repositories may include broadly applicable, high-quality backbone scaffolds. The backbones are next docked to the target, and comprehensive design is performed on residue sequences and conformations, typically employing algorithms like Rosetta [25], ProteinMPNN [26], and LigandMPNN [27]. In the third step, a range of metrics—such as Rosetta scores (‘ddg’, ‘contact molecular surface’, ‘sasa’, ‘contact patch’) and AF2 initial guess parameters (‘pLDDT’, ‘RMSD’, ‘PAE’, ‘pTM’)—are employed as pre-screening to filter the designs [28–30]. This workflow can be iteratively cycled to incrementally improve design quality. Subsequently, experimental screening is performed to identify candidates with high binding affinity, which are then validated structurally and functionally.

## Deep learning driven advances in de novo protein design

In recent years, deep learning has driven a substantial shift in de novo protein design, with particularly notable progress in the design of binders. For a considerable period, achieving high binding affinity and meaningful in vivo functional performance with de novo designed binders was almost inevitably dependent on subsequent directed evolution and large-scale experimental screening [31, 32]. In this traditional framework, computational design primarily served to generate initial candidate molecules, whereas substantive performance optimization, including affinity enhancement and functional refinement, largely occurred at the experimental stage.

This situation did not undergo a substantive change until recent years. With the open release of AlphaFold2 in 2021 [33], the introduction of AlphaFold and ProteinMPNN into protein sequence design in 2022 [34], and the subsequent public availability of generative models such as RFdiffusion [23], the overall workflow of de novo protein design has been systematically reshaped.

These approaches are all centered on deep learning and operate at different levels, including structure prediction, sequence optimization, and backbone generation. In the current, relatively mature design workflow, researchers typically employ RFdiffusion to generate protein backbones, use ProteinMPNN to optimize amino acid sequences, and apply AlphaFold2 to predict and screen structural plausibility and potential binding conformations. Within this framework, experimental validation of only a limited number of candidate sequences is often sufficient to obtain protein molecules with high binding affinity and well-defined in vivo functionality, thereby substantially reducing reliance on large-scale directed evolution and high-throughput screening [34, 35].

The transformative impact of deep learning on de novo protein design arises from its fundamental differences from conventional physics-based strategies. Traditional approaches exemplified by Rosetta are primarily grounded in explicit energy functions and localized conformational sampling, with structural stability and interaction feasibility assessed through energy minimization procedures [36]. Although these methods are well suited for fine-grained structural refinement, their effective search space is constrained by sampling efficiency and the representational limits of the energy functions. As a result, they often struggle to efficiently explore the highly complex and high-dimensional landscapes that couple protein sequence, structure, and function [37].

In contrast, the effectiveness of deep learning approaches is underpinned by the long-term accumulation of protein sequence and structural data over the past several decades. By extracting latent statistical patterns from large-scale protein sequence and structure datasets, these models achieve a more comprehensive representation of the relationships linking sequence, structure, and function [33, 38].Through this data-driven learning process, deep learning models implicitly capture extensive empirical features of natural proteins, including constraints related to folding behavior, structural stability, and molecular interactions.

As a consequence, the design process is no longer restricted to local energy minimization or limited conformational sampling. Instead, candidate proteins can be generated directly within a substantially expanded design space, yielding structures that more closely resemble naturally foldable and physically realizable conformations [23, 39]. At the same time, structure prediction frameworks represented by AlphaFold demonstrate superior accuracy in the evaluation and prioritization of candidate designs compared with traditional physics-based scoring approaches. This improvement in predictive performance strengthens the assessment of structural plausibility and sequence foldability, thereby increasing the likelihood of identifying high-affinity molecules with only a limited amount of experimental validation [28].

## Functions and clinical applications of de novo protein design

Based on their functional modalities and translational relevance, de novo proteins can be broadly categorized into binders, receptor agonists, vaccine scaffolds, and protein switches (Table 2).

**Table 2:** De Novo designed protein functions and their clinical translational potential

Category		Function	Clinical Translational Potential
Binder	Protein binders	Bind selectively to receptors, enzyme active sites, or other protein surfaces to block interactions between endogenous ligands and their targets, thereby achieving inhibitory or neutralizing effects; can also serve as high-affinity recognition modules for targeted localization or delivery.	In vivo molecular imaging and tracing, target validation, signaling pathway inhibition, selective neutralization of disease-associated proteins, and targeted delivery of therapeutics or diagnostic probes.
	IDRs/peptide binding proteins	Specifically recognize and bind peptide segments or intrinsically disordered regions (IDRs) within proteins; interfering with protein–protein interactions and pathological aggregation processes driven by disordered regions.	Targeting disease pathways driven by IDRs or peptides (e.g., neurodegenerative diseases and cancer), inhibition of aberrant protein aggregation, and detection or diagnosis of peptide hormones or IDR-associated biomarkers.
	Small-molecule binding proteins	Bind selectively to small-molecule drugs, metabolites, or other low-molecular-weight ligands; can function either to neutralize small molecules or as sensing modules that convert ligand-binding events into detectable signals.	Small-molecule drug or toxin neutralization (detoxification), therapeutic drug monitoring (TDM), detection of metabolites or biomarkers, molecular sensing, and point-of-care diagnostics.
Receptor agonists		Activate downstream signaling pathways by binding to and stabilizing active receptor conformations to precisely modulate receptor signaling activity.	Activation of specific receptor-mediated signaling pathways for therapeutic benefit, including metabolic diseases, immunomodulation, and neuroregenerative therapies; biased or selective activation to reduce adverse effects.
Vaccine scaffolds		Serve as structural platforms to present antigens in defined spatial arrangements; enable correct conformational display of antigenic epitopes through genetic fusion, and achieve multivalent antigen presentation when combined with de novo designed protein nanoparticles.	Subunit vaccine development with enhanced immunogenicity and stability, applicable to vaccines targeting viral pathogens, cancer antigens, and other disease-associated epitopes.
Protein switches		Undergo reversible structural or functional changes in response to specific external stimuli or analytes, converting molecular recognition events into readable signal outputs or controllable functional states.	Point-of-care diagnostics and biosensing, as well as environment-dependent regulation of therapeutic functions to improve safety and minimize off-target effects.

**Binders** are designed to bind specific targets. This category encompasses three subclasses. Protein binders are compact, folded proteins that provide high-affinity and high-specificity target engagement, making them suitable for inhibition, neutralization, or targeting applications [31]. Intrinsically disordered regions and peptides binding protein are designed to recognize transient or conformationally heterogeneous epitopes that are difficult to address with conventional antibodies, thereby expanding the druggable proteome [40]. Small-molecule binding proteins are engineered to recognize or encapsulate low–molecular-weight ligands, enabling applications in sensing, detoxification, or modulation of small-molecule bioavailability [41]. These distinctions directly influence their suitability for different therapeutic contexts.

**Receptor agonists** differ fundamentally from binders in that they are designed not only to bind receptors but also to stabilize signaling-competent conformations that activate downstream pathways [42]. This requirement imposes additional structural and geometric constraints but enables precise modulation of receptor signaling compared to natural ligands [43] (See Sect. “Intrinsically Disordered Regions and Peptides Binding Protein”, Receptor Agonists, for design principles.)

**Vaccine scaffolds** function as structural platforms that present antigens in defined spatial arrangements. By genetically fusing scaffold proteins with epitope fragments, these platforms enable the display of antigenic epitopes in their correct conformations [44]. This property is particularly advantageous for discontinuous B cell epitopes, as complex conformational epitopes can be reconstructed using relatively short peptide sequences. Furthermore, incorporation of such epitope-modified scaffolds into de novo designed protein nanoparticles enables multivalent antigen presentation, thereby further enhancing immunogenicity [45].

**Protein switches** are proteins that undergo reversible structural or functional changes in response to specific external stimuli, thereby enabling the transduction and conversion of molecular signals. These proteins can directly translate analyte recognition into quantitative readouts and are compatible with simple, wash-free homogeneous detection formats, making them highly attractive for point-of-care diagnostic applications [46]. In addition, by enabling environment-dependent activation or deactivation, protein switches allow precise spatiotemporal control of therapeutic functions, which is particularly advantageous for improving safety and minimizing off-target effects [47] (Representative working mechanisms are illustrated in Fig. 3).

### Protein binders

Proteins are fundamental mediators of cellular activities, with protein-protein interactions (PPIs) being essential for preserving physiological homeostasis. PPIs underlie key biological processes, including signal transduction, metabolic control, gene regulation, and immune defense; they also contribute to the pathogenesis of protein toxins and viruses [48], and are thus pivotal targets in pharmaceutical research. Compared to monoclonal antibodies, small-molecule binders theoretically possess enhanced capabilities for tissue or tumor penetration and improved binding to hard-to-access epitopes. While this is similar to small antibody fragments like Fab, scFv, and nanobodies that entered clinical trials earlier [49], de novo designed binders may represent a superior choice due to their potentially shorter development timelines, lower cost, and greater stability.

The explosion of protein design models in recent years has made significant breakthroughs in the quality of de novo binder design targeting PPI sites [23, 26, 28, 30]. A key aspect of de novo design is its ability to generate high-affinity binders for concave, convex, and flat epitopes, such as TNF-α (planar), and TGFβRII, CTLA-4, PD-L1 (convex) [34, 50]. Another advantage lies in the design quality and screening efficiency enabled by computational approaches, allowing experimental validation of fewer than 100 sequences in some cases to identify high-affinity binders [34, 35], bypassing the need for animal immunization, massive random library screening, or testing of over 100,000 candidates. A de novo protein design study by Glögl et al. targeting TNFR1 tested just 96 sequences, from which they identified a binder with sub-10 pM affinity [34]. This binder greatly outperforms current TNFR1-targeting monoclonal antibodies (680 pM) and monomeric Fab, scFv, or nanobodies, whose affinities range from 10 to 100 nM [51].

### Intrinsically disordered regions and peptides binding protein

Intrinsically disordered regions (IDRs) are functional protein segments that lack a fixed 3D structure [52]. IDRs are prevalent in the human proteome, with 44% of proteins containing disordered regions over 30 residues [53]. Proteins with such regions, or those entirely disordered, are termed intrinsically disordered proteins (IDPs) [54]. IDRs have demonstrated considerable potential in elucidating disease mechanisms, and in the development of diagnostic and therapeutic strategies. The transactivation domain of c-Myc is disordered and critical for co-factor recruitment [55], while NUPR1, an 82-residue IDP, is involved in chromatin remodeling and pancreatic cancer [56]. In malaria, the central NANP-repeat IDR of the CSP protein is the main immunogenic region and a core element of the RTS, S vaccine [57]. IDRs are also implicated in neurodegenerative diseases: the N-terminal IDR of α-synuclein mediates membrane binding and fibrillization in Parkinson’s [58], and the polyQ-expanded IDR of huntingtin drives aggregation in Huntington’s disease [59].

Targeting monomeric IDRs to block pathological aggregation represents a promising therapeutic approach [60–62]. Nevertheless, the intrinsic structural flexibility of IDRs renders them traditionally classified as undruggable targets. Additionally, the rapid degradation of disordered antigens post-injection significantly hampers antibody development efforts. Furthermore, existing antibodies targeting specific peptides often suffer from limited stability and poor reproducibility [63–67].

De novo designed binding proteins have achieved landmark breakthroughs in this field. Early designs were limited to recognizing peptides and protein fragments that are disordered in solution but adopt defined secondary structures upon receptor binding [68–70]. Although this approach enabled the targeting of biologically important molecules such as parathyroid hormone, glucagon, Tau protein, amyloid-β (Aβ), and serum amyloid A1 [71–74], it inherently relied on predefined target structures, which may not represent the most favorable conformations for binder interaction. This structural assumption-imposed limitations on binder efficacy and target flexibility.

Encouragingly, recent advances in design strategies have begun to overcome the longstanding challenges associated with targeting IDRs characterized by high sequence variability and structural flexibility. For the first time, these innovations have enabled the selective generation of binders based solely on sequence information of IDRs or flexible peptides [40, 75, 76]. This represents a significant expansion of the targetable protein landscape, offering access to a broad spectrum of previously undruggable targets. It is therefore plausible to speculate that these developments could initiate a new wave of therapeutic and diagnostic innovations for the aforementioned diseases.

### Small-molecule binding proteins

Numerous drugs and biomarkers are small molecules (SMs), making their detection crucial for clinical prevention, diagnosis, and monitoring [77–80]. While liquid chromatography–mass spectrometry (LC-MS) is the gold standard [81, 82], its low throughput, complex sample preparation, reliance on specialized equipment and personnel, and long turnaround time hinder routine clinical use [83]. Immunodetection methods offer higher throughput and easier operation, but face challenges due to the limited antigenic epitopes on SMs, which require antibodies with exceptionally high affinity and specificity [84–86]. De novo design of small-molecule binding protein, rather than generating antibodies via conventional methods, offers a novel approach for SM immunodetection.

In recent years, de novo design has made significant advancements in designing small-molecule binding protein. The COMBS (Convergent Motifs for Binding Sites), a physics design algorithm, enabled a major breakthrough by directly generating de novo proteins with high specificity and affinity for SMs [87, 88]. Notably, a recent study have shown that small-molecule binding proteins designed using this algorithm can rapidly clear specific drugs from mice [41].

Nevertheless, this algorithm did not overcome the limited diversity of ligand-binding pockets, which substantially narrows the spectrum of targetable ligands for de novo protein design. The subsequently introduced RFdiffusion All-atom enables the direct generation of binding pockets complementary to small molecule targets, though the resulting proteins often demonstrate limited binding affinity [24]. Shortly thereafter, An et al. released an open-source, general-purpose algorithm integrating DL with Rosetta [29]. This algorithm utilized Hallucination to create various protein backbones shape-matched to small molecules [22], enabling the design of binding proteins for a wide array of ligands, including challenging polar and flexible compounds such as thyroxine and the clinical anti-cancer drug methotrexate.

### Receptor agonists

Compared to binder and inhibitor design, de novo agonist design is more complex, as it requires not only target engagement but also induction of specific conformational shifts in receptor regions. Traditionally, agonist antibody discovery has relied on empirical in vitro and in vivo screening, a labor-intensive and time-consuming process [89]. In contrast, the customizability of de novo design enables precise receptor agonist development, with several successful strategies reported [34, 90–93].

**Fig. 2:**
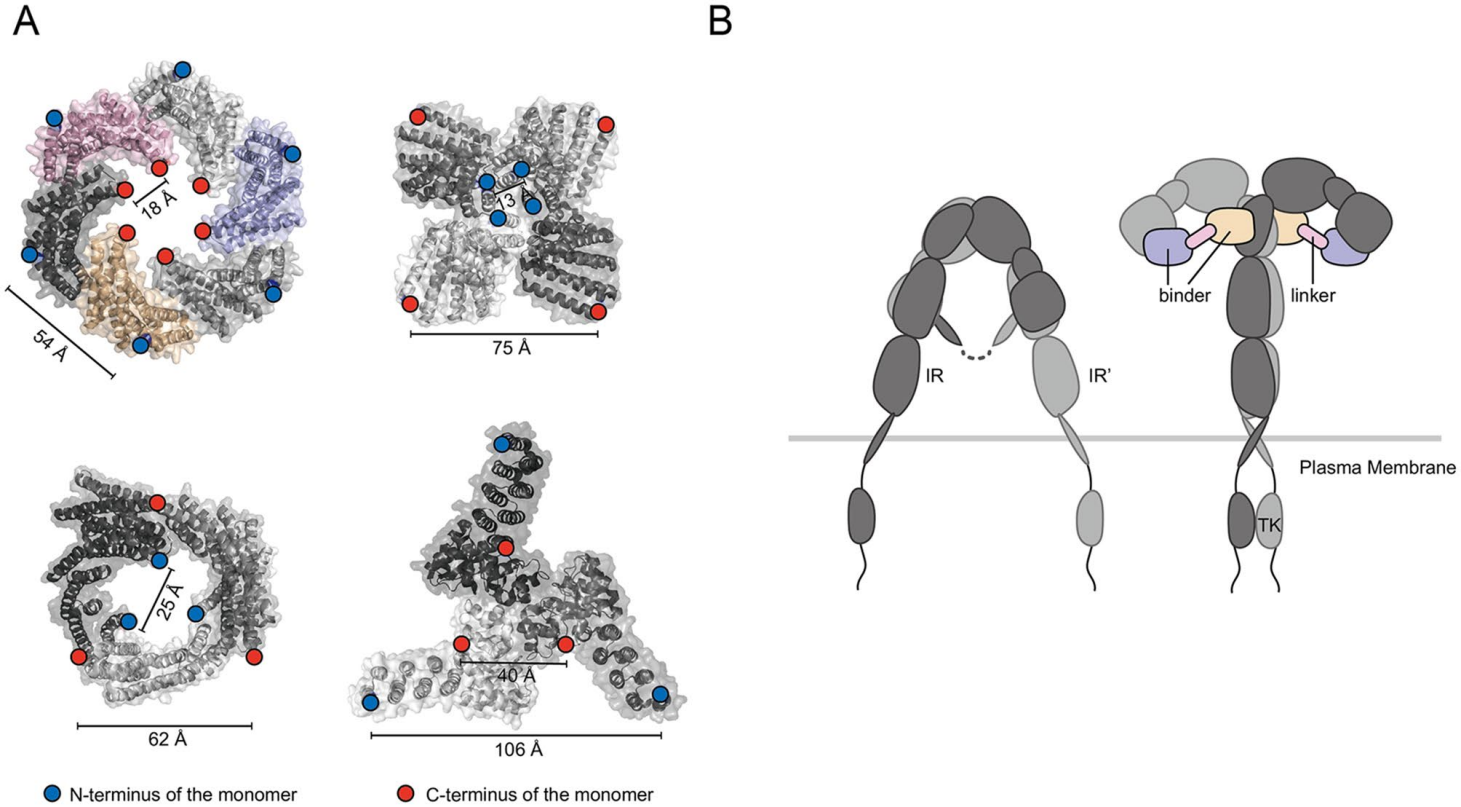
Mechanism of de novo designed agonists. **A**) Examples of de novo designed oligomeric scaffolds; Top left: Protein structure from PDB ID 8F6R. Top right: PDB ID 6XSS. Bottom left: PDB ID 6XNS. Bottom right: PDB ID 6XH5. **B**) The insulin receptor undergoes a conformational change under the action of the de novo designed agonist, reducing the distance between the intracellular tyrosine kinases. Structural illustrations are produced with PyMOL [206]

For a single receptor, one direct strategy is to design a binder that stabilizes its active conformation [90]. For receptors requiring multivalent oligomerization or inter-receptor interactions, two main strategies are applied. One involves integrating ligands into various de novo designed oligomeric scaffolds to fine-tune inter-ligand spacing (Fig. 2A), thereby promoting receptor clustering and initiating valency-dependent signaling [34, 91–93]. For instance, Glögl et al. developed a de novo CD137 agonist [34]. CD137, a T cell costimulatory receptor, must engage its ligand CD137L expressed on APCs in a specific geometric arrangement to activate downstream signaling [94–96]. However, soluble CD137L lacks the structural stability for effective receptor clustering. To overcome this, researchers fused CD137L to the termini of oligomeric protein scaffolds, creating ligand assemblies with defined spatial configurations.

Another approach involves using a binder-linker system to stabilize the receptor’s active state. A notable example is a de novo designed agonist targeting the insulin receptor (IR) [42]. IR functions as a disulfide-linked dimer, with insulin binding to two extracellular sites [97, 98], inducing conformational changes that bring the intracellular tyrosine kinase domains into proximity and activate signaling [99]. This effect can be replicated by designing two site-specific binders connected by a linker of appropriate length and rigidity (Fig. 2B) [42]. In vivo studies show that the de novo agonist exhibits enhanced thermal stability and prolonged glucose-lowering effects compared to insulin. Furthermore, due to their non-overlapping binding residues with endogenous insulin, these agonists can activate IR mutants with impaired insulin binding [100–103].

Overall, our detailed discussion of agonist design highlights the high customizability offered by de novo design. Furthermore, this approach enables the creation of agonists with improved serum stability and target specificity [43, 104, 105]. For natural ligands prone to degradation by serum proteases, de novo mimics offer greater resistance to proteolysis and enhanced signaling efficacy [106]. Improved selectivity can also help minimize adverse effects. For instance, de novo NGF mimics selectively activate TrkA without engaging p75^NTR^, thereby preserving regenerative function while reducing pain sensitization [43].

### Vaccine scaffolds

Vaccines are among the most cost-effective tools for combating infectious diseases, cancer, and chronic conditions [107–111]. Various types exist, including inactivated, attenuated, viral vector, DNA, RNA, and subunit vaccines [112]. Subunit vaccines are favored for their high safety and low adverse event rates. As of March 30, 2023, 183 COVID-19 vaccines were in clinical trials, with subunit vaccines comprising the largest proportion (36%) [113]. However, traditional recombinant protein vaccine development relies on generating large antigen libraries and empirical screening, which is labor-intensive and inefficient, especially since most B-cell epitopes are conformational and discontinuous. These structural challenges complicate rational design.

In contrast, de novo designed scaffolds can maintain the structural integrity of discontinuous epitopes [44] and allow coordinated presentation of multiple epitopes [114, 115], thereby enhancing vaccine stability and immunogenicity. De novo designed scaffolds fused with antigen fragments can generate stable and soluble epitope vaccines, such as those based on the HIV gp120 epitope [116] and noncontiguous epitopes of the RSV fusion protein [117, 118]. A notable case is a syphilis vaccine [119], where challenges in handling outer membrane antigens were addressed by designing a scaffold that mimics membrane support, preserving the antigen’s extracellular conformation.

A prominent example of multivalent antigen presentation is a clinically approved SARS-CoV-2 nanoparticle subunit vaccine based on a self-assembling protein scaffold [120, 121]. The vaccine employs a protein nanoparticle composed of 120 scaffold subunits that spontaneously assemble into a stable icosahedral architecture. By genetically fusing the receptor-binding domain (RBD) to selected scaffold subunits, the assembled nanoparticle achieves highly ordered, multivalent antigen display, resulting in neutralizing antibody titers approximately tenfold higher than those elicited by the prefusion-stabilized spike protein [45].

### Protein switches

Protein switches hold considerable promise for clinical point-of-care diagnostics and therapeutic regulation owing to their ability to directly convert molecular recognition events into functional outputs. Previous research on protein switches has largely been limited to the modification of naturally occurring proteins [122], whereas the identification of native proteins capable of undergoing analyte-induced conformational changes remains highly challenging. De novo protein design overcomes these limitations by enabling the creation of proteins from first principles, thereby allowing the development of modular, programmable, and tunable protein switches with customized sensing and regulatory functions.

**Fig. 3:**
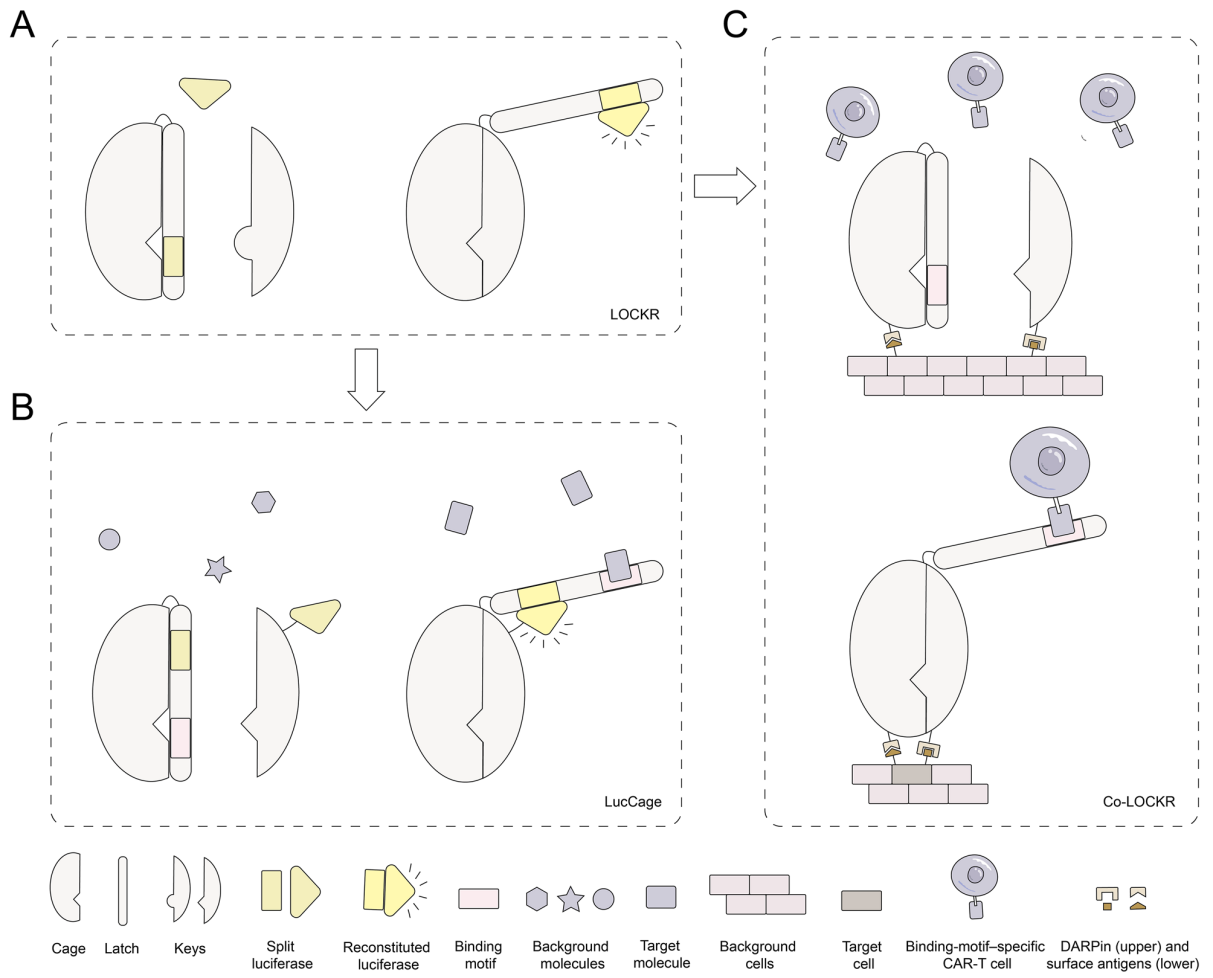
The Latching Orthogonal Cage–Key protein (LOCKR) and its derived protein switch systems. **A**) LOCKR, a switchable protein system that becomes activated in response to a specific key peptide. **B**) LucCage, a biosensing platform designed to recognize specific target molecules. **C**) Co-LOCKR, a switch activated upon binding to a defined set of cell surface antigens, applicable in CAR-T cell targeting

A prominent example of de novo designed protein switches is the LOCKR (Latching Orthogonal Cage–Key protein) system [123] (Fig. 3A), a modular, tunable, and generalizable design composed of a six-helix bundle and a helical peptide known as the “key”. The key peptide can replace a specific helix within the six-helix bundle and expose a signal located on the displaced helix. By making only minor modifications to functional segments and adjusting affinity, the LOCKR system can be applied to engineer diverse biosensors, known as LucCage system (Fig. 3B) and logic gates targeting surface antigen combinations called Co-LOCKR (Fig. 3C) [47, 124].

They show extensive potential in clinical applications, such as targeted protein degradation and localization [123], intracellular signal tracking [125], feedback control [126], minimizing on-target off-tumor responses [127], and the detection of viral proteins, bacterial toxins, cardiac injury markers, antibodies [124], and peptide hormones [68]. However, the LucCage system is not applicable to small-molecule biosensing—a gap that can be addressed using chemical-induced dimerization (CID) systems and ligand-gated channels [29, 128, 129]. Additionally, some de novo designed protein switches serve as sensitive detectors of zinc, calcium ions and pH variations [130–132].

## Challenges in clinical translation and potential solutions

Although a large proportion of de novo designed proteins remain outside clinical development, existing evidence suggests that nearly 90% of drug candidates entering phase I clinical trials ultimately do not succeed [133]. Accordingly, early and systematic evaluation of the clinical translational challenges associated with de novo designed proteins is essential, as it can provide anticipatory insights and strategic guidance for subsequent clinical development.

In light of the relatively scarce clinical translational experience with de novo designed proteins, and considering that their clinical development often initiates with technically established and structurally simple binders, this review integrates, in certain sections, progress in non-antibody scaffolds that are highly comparable to binders with respect to stability, solubility, molecular weight, and production modalities, such as DARPins, Affimers, Affibodies, and Fynomers [134–136]. We anticipate that the accumulated long-term development experience with these molecules will offer valuable guidance for assessing and optimizing the clinical feasibility of de novo designed proteins, and will further promote their rational refinement and translational implementation.

### PK/PD differences between binders and monoclonal antibodies

De novo designed proteins, especially binders, generally exhibit molecular weights of 6–12 kDa [137], markedly lower than those of monoclonal antibodies, which are ~ 150 kDa and possess an Fc domain. Such fundamental structural differences result in marked differences in pharmacokinetic (PK) and pharmacodynamic (PD) characteristics between the two classes.

As proteins smaller than ~ 30 kDa are subject to rapid renal clearance through glomerular filtration, unmodified de novo designed binders generally display very short systemic half-lives, on the order of ~ 10 min in vivo [138, 139]. In contrast, the large molecular size of monoclonal antibodies precludes renal clearance, and FcRn (neonatal Fc receptor)–mediated recycling protects them from lysosomal degradation, enabling sustained systemic exposure with circulation half-lives commonly lasting for weeks [140].

In certain cases, a small molecular size coupled with a short circulation half-life may represent a distinct advantage. For instance, in oncologic molecular imaging, monoclonal antibodies frequently exhibit elevated background signals as a result of restricted tissue penetration and extended systemic retention, which can impair lesion detectability [141, 142]. In contrast, de novo designed binders exhibit faster tumor penetration and more rapid clearance from non-target tissues, which increases tumor–background contrast and consequently improves image resolution and overall diagnostic accuracy [143].

In the context of immunomodulatory treatment, drug circulation half-life represents a key determinant, especially for indications requiring short-term or finely tuned modulation of immunosuppressive intensity. In these clinical contexts, short-acting immunomodulatory agents may provide increased flexibility with respect to safety and dosing control relative to long-acting therapeutics [144]. As an illustration, in some oncologic, autoimmune, or severe infectious disease settings, prolonged exposure to long-acting immunomodulatory drugs can elevate the risk of infection-associated complications [145–148]. While the clinical implications of short half-lives require further validation, they may offer therapeutic advantages in contexts requiring transient target engagement and reduced systemic exposure [149].

Nevertheless, an overly short systemic half-life can constrain the use of binders in chronic indications or treatment settings demanding prolonged target suppression. To address this challenge, a range of half-life extension strategies for small protein drugs have been implemented in de novo protein design, such as albumin-binding approaches and PEG conjugation. For instance, fusion of a de novo designed TcsL inhibitor with the albumin-binding domain M79 enabled reversible binding to serum albumin, thereby substantially extending its in vivo circulation time [35]. Likewise, site-specific introduction of a cysteine residue at position 62 of the de novo designed cytokine mimetic Neo-2/15 enabled conjugation to a single 40 kDa linear PEG, resulting in a long-acting variant with a markedly prolonged in vivo half-life [150].

### Immunogenicity

Immunogenicity constitutes a central consideration in the safety evaluation of biologic therapeutics and must be systematically assessed [151]. Although de novo designed proteins generally do not share sequence homology with endogenous human proteins, thereby theoretically lowering cross-reactivity risk, their non-native sequences can still induce anti-drug antibodies via neo–T cell epitopes or aggregation-associated structures. These immune responses can affect pharmacokinetic and pharmacodynamic properties and may additionally lead to hypersensitivity reactions, immune complex–mediated toxicity, or immune imbalance during prolonged dosing. Accordingly, comprehensive immunogenicity assessment is essential.

Across the majority of preclinical animal models, de novo designed proteins show low immunogenicity or induce only minimal adverse reactions (Table 3). These favorable immunological properties likely reflect multiple contributing factors, including the small molecular size, structural simplicity, and high stability of many de novo designed proteins [152], which reduce cellular uptake and antigen processing, as well as their high solubility and compact conformations that substantially limit protein aggregation—a well-established driver of immune activation [153]. These structural and physicochemical features limit antigen processing and MHC class II–dependent presentation by antigen-presenting cells, resulting in reduced T cell–mediated immune responses [154].

**Table 3:** De Novo designed proteins in animal experiments, clinical trials, and approved for clinical application

Design	Applications (in animal)	Animal model	route of administration	Tolerability and immunogenicity in animals	Clinical Trials identifiers (Phase, Recruitment status)	ref.
Mimics of IL-2/IL-15	anti-tumor	mouse models of melanoma /colon cancer	intravenous injection	reduced toxicity and undetectable immunogenicity	NCT04659629(Ⅰ, Completed)	[104]
IL-23R binder	inflammatory bowel disease	mouse model of colitis	oral administration	low predicted immunogenicity	-	[192]
Anti-Three-finger toxins (3FTx) binder	snakebite envenoming	murine α-bungarotoxin poisoning model	intraperitoneal injection	no negative effects	-	[195]
SARS-CoV-2 RBD binder	viral infection	mouse	intranasal administration /intraperitoneal injection	1/10 developed IgG antibodies against binder	-	[196]
Endocytosis-inducing proteins	targeted receptor degradation (e.g., PD-L1)	BALB/c nude mouse tumor model	intratumoral injection	body weight assessment indicated good tolerability	-	[197]
αvβ6 binder	pulmonary fibrosis (PF)	bleomycin-induced pulmonary fibrosis mouse model	intraperitoneal injection / oropharyngeal administration	-	-	[139]
IL-21 mimics	anti-tumor	mouse models of B16F10 melanoma/mc38 adenocarcinoma	Intraperitoneal injection	5/30 developed antibodies against binder	-	[106]
Pan-Ebolavirus Nanoparticle Vaccine	vaccine	mouse/guinea pig	subcutaneous injection	-	-	[198]
SARS-CoV-2 Nanoparticle Vaccine *	vaccine	mouse/rhesus macaque	intramuscular injection	anti-NP scaffold response observed	NCT05007951(Ⅲ, Completed)	[45, 199]
RSV Nanoparticle Vaccine	vaccine	mouse/rhesus macaque	injection	anti-NP scaffold response observed; reduced with increasing antigen display density	-	[158]
MERS-CoV RBD binder	viral infection	mouse	intranasal administration	-	-	[200]
IR agonist	diabetes	healthy mouse / diet-induced obesity mouse	intraperitoneal injection	-	-	[42]
TcsL (lethal toxin) inhibitor	P. sordellii infection	mouse model of P. sordellii toxic shock	intraperitoneal injection	-	-	[35]
SARS-CoV-2 variants inhibitor	viral infection	Beta/Delta/Omicron-infected K18-hACE2 mouse	intranasal administration	-	-	[201, 202]
Synthetic Intramembrane Proteolysis Receptor	CAR-T	mouse models of A375 melanoma	retro-orbital injection	body weight assessment indicated good tolerability	-	[203]
EGFR binder	CAR-T	healthy mouse / orthotopic xenograft mouse model of glioblastoma	intraperitoneal / intracranial injection	no apparent toxicity or side effects	-	[204]
CD276 binder	CAR-T	healthy mouse / orthotopic xenograft mouse model of glioblastoma	intraperitoneal / intracranial injection	no apparent toxicity or side effects	NCT06691308(Ⅰ, not yet recruiting)	[204]
D-protein small molecule binders	drug clearance	mouse	intraperitoneal injection	undetectable immunogenicity	-	[41]
Integrin α5β1 agonist	tissue regeneration	rabbit	biomaterial coating for implants	no local inflammation or foreign body response		[205]

* Approved for clinical application

Nevertheless, favorable physicochemical properties alone cannot fully eliminate immunogenicity risk. Taking DARPins as an example, despite their high solubility, stability, and low propensity for aggregation, which are thought to limit T cell independent immune responses [155], a DARPin therapeutic was ultimately withdrawn following the development of anti-drug antibodies and associated intraocular inflammatory reactions in clinical settings [156].

In addition, despite promising outcomes observed for numerous de novo designed proteins in preclinical animal studies, few have advanced to clinical evaluation or achieved approval for clinical use (Table 3). Thus far, the only example is the de novo designed cytokine mimetic Neo-2/15 reported in 2019, which showed low immunogenicity in human studies and offers initial yet limited evidence supporting clinical translation [157]. Meanwhile, larger and more structurally complex de novo designed proteins, including nanoparticle vaccine scaffolds, may carry an increased risk of eliciting scaffold-specific antibody responses [158], a topic that is addressed in greater detail in Sect. “Clinical feasibility of nanoparticle vaccines and oligomeric scaffolds”, “Clinical feasibility of nanoparticle vaccines and oligomeric scaffolds.”

Immunogenicity is highly unpredictable and influenced by multiple factors, including formulation properties, routes of administration, and patient disease status [159], while available strategies to mitigate immunogenicity remain limited. Recently, immunoinformatic prediction and redesign of putative T cell epitopes have been explored to mitigate immune activation [160], but such approaches are largely grounded in sequence-level and MHC binding models and therefore incompletely reflect in vivo immunological complexity. Meanwhile, de novo design of heterochiral proteins has been suggested as a potential means to lower immunogenicity by impairing immune recognition [41, 161], yet its scalability and clinical translatability remain significantly constrained.

Overall, evaluating and mitigating immunogenicity continues to represent a major unresolved challenge in de novo protein design and across the broader biologics development pipeline.

### Chemistry, manufacturing, and controls (CMC) considerations

Before clinical testing, biologic candidates must comply with CMC requirements through comprehensive evaluation of their physicochemical or biological characteristics, manufacturing processes and controls, quality standards, and stability data [162, 163].

De novo designed proteins can be co-optimized for expression, solubility, and stability at the sequence design stage [164] and are often compatible with mature, cost-effective expression systems such as Escherichia coli, thereby offering potential advantages in manufacturability and scalability.

Nevertheless, these benefits are primarily derived from laboratory-scale studies, and further process optimization is required in real-world manufacturing and clinical development to address scale-up, facility transfers, and increasingly stringent GMP standards [165]. As a result, systematic assessment of long-term stability, formulation compatibility, batch consistency, and process scalability remain essential.

Efficient developability assessment at an early stage is essential for advancing chemistry, manufacturing, and controls (CMC) processes within acceptable cost and time frames [166]. This approach centers on systematic assessment of clinically relevant properties, including stability, immunogenicity risk, toxicological liabilities, and expression levels, at the candidate screening and optimization stages. Integrating such considerations into molecular design or early in silico assessment stages could enhance screening efficiency and mitigate time and resource demands during subsequent CMC development.

Moreover, drawing on prior experience with non-antibody scaffold proteins [167], and partnering with experienced manufacturing groups and mature technology platforms can accelerate the development and validation of CMC frameworks.

### Toxicology

From a toxicological perspective, biologics’ adverse reactions are primarily driven by immunogenicity and exaggerated pharmacological effects [149, 168]. Immunogenicity-related risks have been discussed in Sect. 5.2, and exaggerated pharmacology generally arises from overmodulation of the target and involves mechanism-related risks that are somewhat predictable [169].

Based on this, risk management often relies on mechanism-guided approaches coupled with exposure management, for example by setting initial doses according to the minimum anticipated biological effect level (MABEL) and using PK/PD relationships and biomarker monitoring to restrict early exposure and lower the risk of adverse reactions [170].

Moreover, the small molecular size of most de novo designed proteins leads to predominant renal clearance via glomerular filtration and therefore requires careful consideration of renal exposure and potential accumulation, particularly in molecular imaging applications. While clinical evidence for de novo designed proteins is still scarce, existing studies indicate that small protein or scaffold-based radiolabeled imaging probes may be reabsorbed in the proximal tubules, leading to renal retention of radioactive labels and associated nephrotoxic risk.

To mitigate these risks, some engineering approaches have been developed, such as replacing residualizing with non-residualizing radiolabels to enhance tubular efflux and hasten renal signal decay [171, 172], or introducing site-specific amino acid substitutions to increase molecular surface hydrophilicity and thereby decrease renal retention [134].

### Clinical feasibility of nanoparticle vaccines and oligomeric scaffolds

De novo designed nanoparticle vaccines and oligomeric scaffolds exhibit significant advantages in improving antigen presentation and multivalent engagement, but their clinical translatability remains to be cautiously assessed.

Evidence suggests that excessive antigen density on nanoparticle surfaces can limit epitope accessibility and reduce effective immune recognition of key antigenic sites [173], and excessive steric hindrance may disrupt proper nanoparticle assembly, leading to partially assembled structures. In addition, nanoparticles and oligomeric scaffolds may also face risks of in vivo disassembly [174].

Furthermore, scaffolds can provoke nonspecific anti-scaffold immune responses, potentially associated with their molecular size and structural complexity. In general, increased molecular size and structural complexity are associated with a greater propensity for immunogenicity [175]. Fortunately, higher surface epitope density on nanoparticles is associated with reduced anti-scaffold immune responses [158].

To address these issues, several strategies may be informative. For instance, optimizing antigen spacing on nanoparticles can mitigate crowding effects while maintaining the geometry necessary for multivalent B-cell engagement, which may enhance antigen presentation; in certain immunogens, refining the geometry of epitope display can further improve accessibility [176]. Moreover, increasing the affinity and shape complementarity at subunit interfaces may serve as a potential strategy to reduce the risk of disassembly in vivo.

## Current limitations and future challenges in de novo protein design

### Limitations of deep learning-based design

Although deep learning has markedly improved the efficiency of de novo protein design in structure generation and sequence optimization, its performance is still limited by dataset bias, restricted generalization capacity, and inadequate representation of protein conformational dynamics and context dependence.

Protein function often depends on conformational changes and associated dynamic processes [177], whereas most current models are trained predominantly on static crystal structures and therefore fail to represent the flexibility, conformational heterogeneity, and functional motions of proteins in physiological environments [14]. These limitations are particularly pronounced for membrane proteins, macromolecular complexes, and intrinsically disordered proteins, underscoring the need to integrate multimodal experimental data and explicit physical constraints beyond static structure-based models [178].

Meanwhile, because the PDB largely archives only successfully folded proteins, it inherently introduces survivor bias, preventing models from accessing informative negative examples associated with failed designs. With the advancement of automated wet-lab platforms [179], establishing closed-loop “design–experiment–feedback” workflows may represent a key direction for improving the reliability and generalizability of protein design [180].

Moreover, current machine learning approaches to protein structure prediction can yield nonphysical conformations, such as segment overlaps, steric clashes, and unrealistic bond parameters. To address this challenge, incorporating physical principles into modeling frameworks is considered an effective strategy. As an example, the Boltz-1x framework incorporates a Boltz-steering strategy, whereby physics-informed energy constraints are imposed at inference time to effectively reduce the incidence of such nonphysical conformations [181].

### Barriers to successful in vivo translation

Despite strong functional activity observed for many de novo designed proteins in vitro [34, 144], successful translation to in vivo therapeutic efficacy remains challenging. This is primarily because in vitro systems reflect only partial biological phenomena and inadequately model the complexity of in vivo physiology and pathology.

Several factors contribute to this gap. For certain diseases, such as cancer [182], intracerebral hemorrhage [183], and Alzheimer’s disease [184], existing in vitro or simplified cellular models fail to accurately reconstruct core pathophysiological characteristics. Moreover, therapeutic efficacy in vivo is influenced by multiple factors, including dosing, delivery route, pharmacokinetics, immunogenicity, and stability, any of which may cause candidates that are effective in vitro to rapidly lose efficacy in vivo. Additionally, conformational dynamics, nonspecific interactions, and compensatory pathway activation in vivo can further compromise the persistence of functional efficacy.

Notably, in some cases, limited in vivo translatability may primarily reflect insufficient biological validity of the chosen target. Prior experience in biologic drug development provides clear examples, such as overly high binding affinity leading to a “binding-site barrier” that restricts penetration into solid tumors [185]. Within highly heterogeneous tumors, excessive target specificity can drive antigen escape and facilitate the development of therapeutic resistance [186]. Moreover, intervention at highly networked regulatory hubs often triggers systemic effects that are difficult to predict and control [187].

Accordingly, with rising design efficiency and falling costs, the success of in vivo translation for de novo designed proteins may depend less on molecular design feasibility and more on the depth of understanding of disease biology.

### Challenges in designing β-rich proteins

Currently, de novo protein design has largely focused on all-α and mixed α/β architectures, with comparatively slower advances in β-rich protein design [188]. This difference fundamentally arises because structurally viable β-sheet proteins do not follow simple idealized geometries. Extensive structural analyses reveal that β-sheet architectures in nature commonly exhibit non-ideal features, including β-bulges, local sheet twisting, and edge defects. These structural “imperfections” are not incidental but play important structural and functional roles, such as relieving backbone strain and preventing unchecked extension of β-sheets along the backbone, thereby reducing the risk of misfolding and aggregation [189, 190].

During de novo design, researchers have attempted to partially alleviate backbone strain by introducing structural features that deviate from ideal β-geometry, such as β-bulges or glycine-induced local kinks [25]. Nonetheless, with increasing β-strand count and topological complexity, locally reasonable design rules frequently give rise to significant global backbone strain at the tertiary structure scale. Such strain prevents the designed topology from corresponding to the lowest free-energy state, promoting spontaneous rearrangements such as β-strand swapping and substantially lowering the likelihood of successful design [191].

### Post-design reliance on directed evolution

With continued advances in de novo protein design, dependence on directed evolution for achieving high-affinity binding molecules is generally declining, especially in the context of protein binder design. In early binder development, directed evolution or extensive screening was largely unavoidable, while computational design mainly provided starting backbones or low-affinity lead candidates. More recently, advances in deep learning–based structure generation and sequence optimization have allowed certain designs to reach nM to pM binding affinities with little additional screening.

Nevertheless, directed evolution is still hard to dispense with for designs that demand additional functionality or adaptation to specific environments. As an illustration, a de novo designed IL-23R binding protein for the treatment of colitis was refined beyond computational design using site-saturation mutagenesis and combinatorial library screening [192]. Importantly, this approach focused not on further enhancing affinity, but on tailoring acid stability, and protease resistance for gastrointestinal administration, ultimately conferring oral bioavailability.

Indeed, in complex design settings, directed evolution and de novo protein design are frequently complementary. In some situations where natural protein templates or established functional prototypes are lacking, directed evolution by itself may struggle to generate functional starting variants; in contrast, de novo design can produce artificial proteins with baseline activity, offering a feasible foundation for downstream experimental optimization [193].

Meanwhile, directed evolution coupled with high-throughput screening yields extensive variant datasets and mutation–function relationships that may be used to calibrate, fine-tune, and benchmark models. Within antibody development, industry efforts have combined rapid experimental platforms with machine learning to enable antibody sequence design and optimization through iterative design–build–test workflows [194].

While directed evolution datasets are typically context-dependent and task-specific and thus unsuitable for training general structure generators, they are particularly valuable for refining sequence–function models, correcting scoring functions, and informing iterative design. Over time, dependence on directed evolution is likely to evolve into a feedback mechanism within integrated design–experiment loops, rather than continued reliance on extensive empirical screening.

## Outlook

De novo protein design has emerged as a powerful platform for generating proteins with highly customizable structures and functions, with clear advantages over conventional discovery methods in flexibility, modularity, and scalability. Potential clinical uses include high-affinity binding proteins, receptor agonists, vaccine scaffolds, and protein switches, and may enable the targeting of protein surfaces and functional sites traditionally considered undruggable.

Nevertheless, translating de novo designed proteins into the clinic extends beyond computation or structure alone and represents a systemic challenge encompassing pharmacology, pharmacokinetics, immunology, and manufacturing considerations. Critical advances will require close coordination between computational design, experimental testing, engineering refinement, and clinical requirements. With early integration of clinical feasibility and ongoing optimization of translational attributes throughout development, de novo protein design holds promise for advancing from proof-of-concept to real-world clinical application.

## Data Availability

All data supporting the findings of this study are included in the manuscript.
